# Diagnostic accuracy and outcomes of lymph node staging in intermediate‐risk prostate cancer

**DOI:** 10.1111/bju.70155

**Published:** 2026-01-23

**Authors:** Christopher Kniep, Tobias Maurer, Ben Frederik Hartwieg, Philipp Mandel, Mike Wenzel, Fabian Falkenbach, Kristian Krpina, Lars Budäus, Alexander Haese, Georg Salomon, Thomas Steuber, Markus Graefen, Derya Tilki, Felix Preisser

**Affiliations:** ^1^ Martini‐Klinik Prostate Cancer Centre University Hospital Hamburg Eppendorf Hamburg Germany; ^2^ Department of Urology University Hospital Hamburg‐Eppendorf Hamburg Germany; ^3^ Department of Urology University Hospital Frankfurt Frankfurt am Main Germany; ^4^ Department of Urology, Faculty of Medicine, Clinical Hospital Centre Rijeka University of Rijeka Rijeka Croatia

**Keywords:** staging accuracy, prostate cancer, intermediate risk, PSMA‐PET, computed tomography, magnetic resonance imaging

## Abstract

**Objective:**

To evaluate the diagnostic performance of different staging modalities and oncological outcomes in patients with intermediate‐risk (IR) prostate cancer (PCa) undergoing radical prostatectomy (RP) with pelvic lymph node dissection (PLND).

**Patients and Methods:**

Patients with IR PCa who underwent RP and PLND between 2015 and 2021 were retrospectively analysed. Patients who had received neoadjuvant hormone therapy were excluded. The effectiveness of computed tomography (CT), magnetic resonance imaging (MRI), and prostate‐specific membrane antigen‐positron emission tomography (PSMA‐PET) in detecting lymph node invasion (LNI) was assessed. Kaplan–Meier analysis was used to evaluate biochemical recurrence‐free and metastasis‐free survival.

**Results:**

Among 8043 patients with IR PCa undergoing RP with PLND, 624 (7.8%) had LNI. PSMA‐PET was performed in 400 patients: six true positives, 40 false negatives, 14 false positives, and 340 true negatives. CT was used in 2079 patients: two true positives, 228 false negatives, seven false positives, and 1842 true negatives. MRI was performed in 148 patients: one true positive, 11 false negatives, and 136 true negatives, with no false positives. Sensitivity was highest for PSMA‐PET (13%), followed by MRI (8.3%) and CT (0.9%). Negative predictive values were 92.5% for MRI, 89.5% for PSMA‐PET, and 89% for CT. Patients with negative PSMA‐PET findings had significantly better biochemical recurrence‐free and metastasis‐free survival than those with suspicious findings on PSMA‐PET.

**Conclusions:**

All evaluated staging modalities demonstrated limited sensitivity in detecting LNI in patients with IR PCa, including PSMA‐PET. Given the poor diagnostic performance of conventional imaging, such methods may be omitted in this setting. PSMA‐PET may still be considered selectively, as it provides modest sensitivity and prognostic value, although its role remains limited.

**Abbreviations**
ADT: androgen deprivation therapy
BCR: biochemical recurrence
cT: clinical T stage
IQR: interquartile range
IR: intermediate risk
ISUP: International Society of Urological Pathology
LNI: lymph node invasion; mi, molecular imaging
NCCN: National Comprehensive Cancer Network
NPV: negative predictive value
PCa: prostate cancer
PLND: pelvic lymph node dissection
pN: pathological N stage
PPV: positive predictive value
PSMA‐PET: prostate‐specific membrane antigen positron emission tomography
RP: radical prostatectomy

## Introduction

Prostate cancer (PCa) remains one of the most commonly diagnosed malignancies in men worldwide, with varying prognoses depending on the stage and risk classification at diagnosis. Among patients with intermediate‐risk (IR) PCa—a category that represents a significant proportion of newly diagnosed cases—treatment decisions often involve a delicate balance between oncological control and quality of life [1]. Radical prostatectomy (RP) continues to be a widely used curative treatment option for this group [1]. However, the optimal approach for lymph node staging in patients with IR PCa remains an area of ongoing debate. The PCa guidelines from the European Association of Urology (EAU) give a weak recommendation for patients with International Society of Urological Pathology (ISUP) Grade Group 3 disease to perform a prostate‐specific antigen‐positron emission tomography(PSMA‐PET)/CT if available to increase accuracy or at least a cross‐sectional abdominopelvic imaging [2]. Conversely, the AUA practical guidelines for PCa recommend that clinicians should not routinely perform abdominopelvic CT in asymptomatic patients with low‐risk or IR PCa (expert opinion) due to the low number of events in this population [3]. Similar to the inconsistent guidelines’ recommendations for conventional imaging, a recently published review article from Carll et al. [4] summarises the very inconsistent recommendations for the use of PSMA‐PET in patients with IR PCa between various societies.

One previous study by Risko et al. [5] demonstrated that the implementation of criteria for CT imaging that includes PSA value of >20 ng/mL, or Gleason Score ≥8 or locally advanced disease (interpreted as clinical T stage [cT]3/4) would ensure that CT scans are performed for almost all men who would have studies positive for metastases, with an estimated missed positive rate of <1%, while at the same time reducing the total number of staging evaluations by >25%.

On the other hand, accurate lymph node staging may impact both, prognostication and treatment planning. The presence of lymph node metastases is associated with worse oncological outcomes, and identifying nodal involvement can influence decisions regarding adjuvant therapies [6]. A variety of lymph node staging procedures exist, ranging from cross‐sectional imaging procedures to pelvic lymph node dissection (PLND). Yet, the clinical utility and comparative effectiveness of these staging strategies in patients with IR PCa are not well established. While an extended PLND may be the most sensitive staging procedure, it is also associated with increased operative time, complications, and healthcare costs, raising questions about its routine use in patients with IR PCa during RP.

Given the heterogeneity within the IR category and the evolving nature of staging technologies, such as the introduction of PSMA‐PET with its superior sensitivity, it is essential to assess the relative performance of available lymph node staging procedures in terms of both diagnostic accuracy and oncological outcomes. Evaluating these procedures can provide valuable insights into their utility in detecting occult metastases, reducing recurrence rates, and improving survival, all while considering the trade‐offs in perioperative morbidity.

This analysis aimed to assess and compare various lymph node staging strategies utilised during RP in patients with IR PCa. Specifically, we analysed their impact on nodal detection rates, biochemical recurrence (BCR)‐free, and metastasis‐free survival. By doing so, we sought to clarify the role of lymph node staging in this patient population. These findings may have significant implications for optimising the use of staging procedures in IR PCa.

## Patients and Methods

### Study Population

**Fig. 1:**
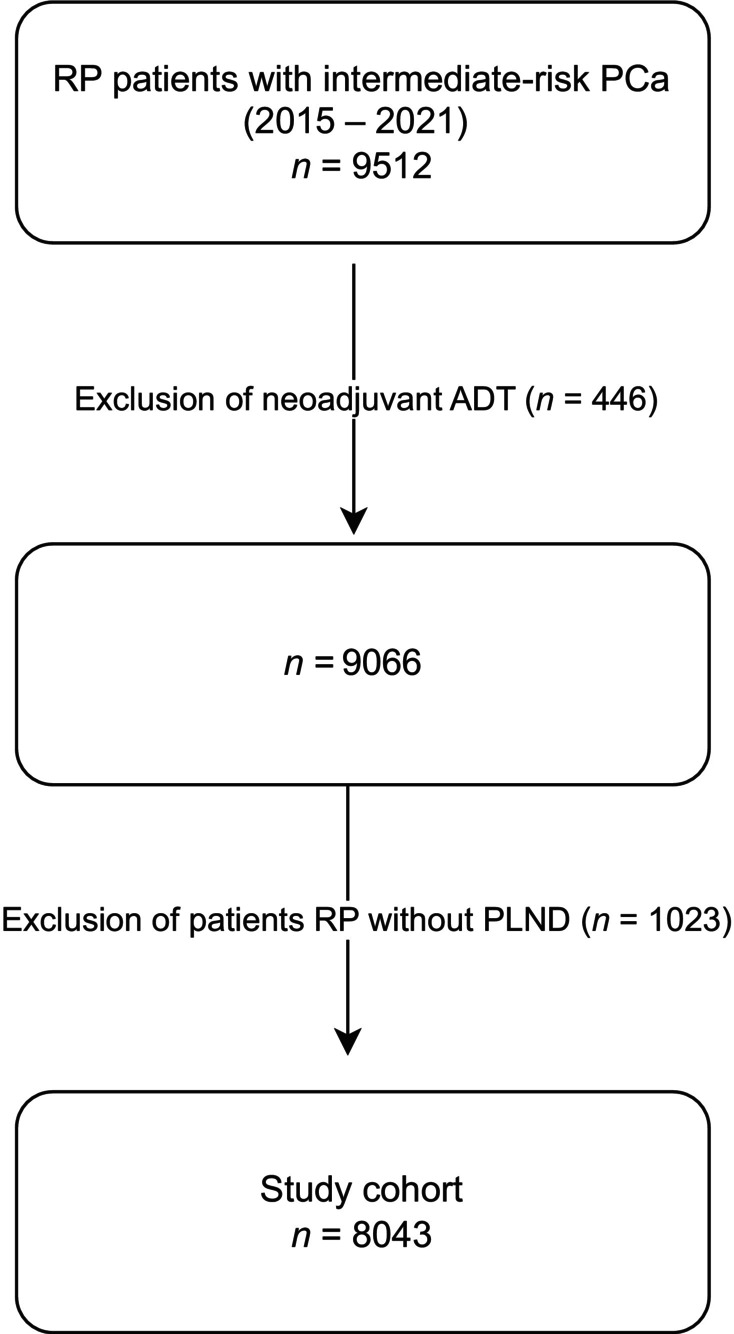
Flowchart depicting the study cohort and patient selection criteria.

After Institutional Review Board approval, patients with IR PCa who underwent RP with PLND (January 2015–December 2021) at the Martini‐Klinik were identified (Fig. 1). During the study span all patients with IR PCa, according to our national guidelines, were candidates for PLND during RP. Only in case of challenging surgical conditions, e.g., extensive fibrotic changes after bilateral inguinal mesh hernia, which would make resection more complex or if the patient refused to have a PLND, was it omitted.

We evaluated the use of different staging techniques for assessing pelvic lymph nodes in patients undergoing RP, including abdominopelvic contrast‐enhanced high‐dose CT, abdominopelvic contrast‐enhanced MRI, or full‐body PSMA‐PET. PSMA‐PET scans were performed with the use of ^68^Ga or ^18^F as tracer. The PET scans were performed in combination with either CT (native, low‐dose or contrast) and/or MRI. All imaging was performed in nuclear medicine and/or radiology institutions throughout Germany.

Indication for primary staging and the chosen modality was performed at the discretion of the treating physician/urologist. We do not request any cross‐sectional imaging for patients with IR PCa prior to RP and imaging was ordered by the patient's resident urologists. Some patients had a CT and MRI (*n* = 20) or combination of CT and PSMA‐PET (*n* = 60). As the indication for the different imaging modalities was unknown, staging findings were separately assessed as our primary study goal was to assess the performance statistics of various staging modalities. For cases involving positive lymph nodes, as a part of our daily clinical evaluation, a centralised radiological and/or nuclear medicine review was undertaken to validate the findings and support surgeons with intraoperative identification of suspicious lymph nodes.

Stratification was performed according to National Comprehensive Cancer Network (NCCN) favourable and unfavourable IR PCa [7]. Favourable IR PCa was defined as patients harbouring only one IR factor. The IR factors were: cT2b–cT2c, ISUP Grade Group 2 or 3, and PSA value 10–20 ng/mL. In addition, patients with favourable disease had only ISUP Grade Group 1 or 2 and <50% biopsy cores positive. Unfavourable IR PCa was defined as patients harbouring two or three of the above‐mentioned IR factors, ISUP Grade Group 3 or ≥50% biopsy cores positive [7].

Patients with neoadjuvant hormone therapy, which might influence pathological evaluation of the pelvic lymph nodes, were excluded from the analysis. This selection criterion yielded 8043 patients, which represented the focus for the current analysis.

### Surgical Approach and Histological Evaluation

Surgery was performed either as an open retropubic or robot‐assisted laparoscopic approach as previously described [8, 9, 10]. In patients where PLND was performed, all patients received an extended PLND [11]. All pathological evaluations were performed, as part of our routine evaluation, by dedicated uropathologists at the Institute of Pathology at the University Medical Center Hamburg‐Eppendorf. Final pathological reports included location and number of positive lymph nodes.

### Follow‐Up

Patient follow‐up comprised periodical PSA testing and postoperative imaging studies, which were performed according to PSA value and further symptoms. BCR was defined as two consecutive PSA values ≥0.2 ng/mL and rising after RP. PSA follow‐up was performed postoperatively by treating urologists. PSA values were obtained individually from patients by the Martini‐Klinik. BCR‐free survival for each patient was recorded in months, starting from the date of RP until the date of BCR. Metastasis‐free survival was defined as no radiological sign of metastasis in further performed imaging studies [12]. Imaging procedures consisted of bone scan and/or CT and/or abdominal MRI and/or ^11^C‐choline PET/CT scan. Time to metastasis was also calculated as the time from RP to metastasis or last follow‐up.

### Statistical Analyses

Descriptive statistics included frequencies and proportions for categorical variables. Medians and interquartile ranges or (IQRs) means and SDs were reported for continuously coded variables. The chi‐square test tested the statistical significance of proportions’ differences. The Welch two‐sample *t*‐test examined the statistical significance of means differences, respectively.

To evaluate the performance of the different staging modalities, fourfold contingency tables were constructed to determine sensitivity, specificity, positive (PPV) and negative predictive values (NPV), and overall accuracy. Subgroups focused on patients with unfavourable and favourable IR PCa only (Figures S1 and S2; Tables S1 and S2).

Uni‐ and multivariable logistic regression models were used to assess the risk of lymph node invasion (LNI) and patients’ tumour characteristics. Adjustment was made for clinical tumour and patient characteristics (age, preoperative PSA value, clinical tumour stage, biopsy ISUP Grade Group, and number of positive cores).

Kaplan–Meier analyses were used to graphically depict BCR‐free and metastasis‐free survival after RP.

The R software environment for statistical computing and graphics (version 4.2.2 for Mac, The R Foundation for Statistical Computing, Vienna, Austria) was used for all statistical analyses. All tests were two‐sided with a level of significance set at *P <* 0.05.

## Results

### Patients’ Characteristics

Of the 8043 included patients with IR PCa (Table 1) 36.7% vs 63.3% had favourable and unfavourable NCCN IR disease. Across the entire cohort, LNI was identified in 7.8% (*n* = 624) of patients undergoing RP and PLND, comprising 3.2% (*n* = 94) of those with favourable and 10.4% (*n* = 530) with unfavourable NCCN IR disease. Patients with LNI had higher PSA values preoperatively (median [IQR] 9.2 [6.2–13] vs 7.2 [5.1–10.5] ng/mL, *P* < 0.001), more frequently belonged to the unfavourable IR group (84.9% vs 61.5%, *P* < 0.001), and more often had ISUP Grade Group 3 (47.8% vs 24.2%, *P* < 0.001), compared to patients without LNI. The median (IQR) number of lymph nodes removed was 14 (9–19) vs 17 (12–24) for patients without vs with LNI (*P* < 0.001). The median (IQR) number of positive lymph nodes in patient with LNI was 1 (1–2) positive node.

**Table 1:** Descriptive characteristics of the patients with IR PCa that underwent RP and PLND, stratified according NCCN risk group and pathological lymph node (pN) status.

Characteristic	Overall, *N* = 8043	Favourable IR, *n* = 2953 (36.7%)	Unfavourable IR, *n* = 5090 (63.3%)	*P*	pN0, *n* = 7419 (92.2%)	pN1, *n* = 624 (7.8%)	*P*
Follow up, months, median (IQR)	48.7 (25.6–73)	48.8 (26–73)	48.7 (25.4–66.7)	0.04	48.7 (25.6–73)	48.7 (25.7–68.8)	0.9
Age, years, median (IQR)	64 (59–68)	63 (58–68)	64 (59–699)	<0.001	64 (59–68)	65 (60–70)	<0.001
Preoperative PSA value, ng/mL, median (IQR)	7.3 (5.2–10.7)	6.4 (4.8–8.6)	8.1 (5.5–11.7)	<0.001	7.2 (5.1–10.5)	9.2 (6.2–13)	<0.001
Lymph nodes removed, *n*, median (IQR)	14 (9–20)	13 (9–19)	14 (9–20)	<0.001	14 (9–19)	17 (12–24)	<0.001
Prostate volume, mL, median (IQR)	29 (22–39)	30 (23–41)	29 (22–38)	<0.001	29 (22–39)	30 (23–40)	0.1
Biopsy cores taken, *n*, median (IQR)	12 (10–13)	12 (10–14)	11 (9–13)	<0.001	12 (10–14)	11 (9–12)	<0.001
Positive biopsy cores, *n*, median (IQR)	4 (3–6)	3 (2–4)	5 (3–7)	<0.001	4 (2–6)	5 (3–8)	<0.001
Clinical tumour stage, *n* (%)							
T1c	5919 (73.6)	2421 (82)	3498 (68.7)	<0.001	5558 (74.9)	361 (57.9)	<0.001
T2a	1488 (18.5)	468 (15.8)	1020 (20)		1336 (18)	152 (24.4)	
T2b	531 (6.6)	26 (0.9)	505 (9.9)		432 (5.8)	99 (15.9)	
Biopsy ISUP Grade Group, *n* (%)							
ISUP 1	628 (7.8)	470 (15.9)	158 (3.1)	<0.001	601 (8.1)	27 (4.3)	<0.001
ISUP 2	5324 (66.2)	2483 (84.1)	2841 (55.8)		5025 (67.7)	299 (47.9)	
ISUP 3	2091 (26)	0 (0)	2091 (41.1)		1793 (24.2)	298 (47.8)	
RP ISUP Grade Group, *n* (%)							
ISUP 1	239 (3)	185 (6.3)	54 (1.1)	<0.001	239 (3.2)	0 (0)	<0.001
ISUP 2	6150 (76.5)	2476 (83.8)	3674 (72.2)		5898 (79.5)	252 (40.4)	
ISUP 3	1486 (18.5)	263 (8.9)	1223 (24)		1180 (15.9)	306 (49)	
ISUP 4–5	162 (2)	25 (0.8)	137 (2.7)		96 (1.3)	66 (10.6)	
Pathological tumour stage, *n* (%)							
pT2	5194 (64.6)	2263 (76.6)	2931 (57.6)	<0.001	5075 (68.4)	119 (19.1)	<0.001
pT3a	2083 (25.9)	588 (19.9)	1495 (29.4)		1858 (25)	225 (36.1)	
≥pT3b	766 (9.5)	102 (3.5)	664 (13)		486 (6.6)	280 (44.9)	
Positive margins, *n* (%)	1329 (16.5)	317 (10.7)	1012 (19.9)	<0.001	1110 (15)	219 (35.1)	<0.001
Adjuvant ADT, *n* (%)	539 (6.7)	78 (2.6)	461 (9.1)	<0.001	305 (4.1)	234 (37.5)	<0.001
Abdominal CT performed for staging, *n* (%)	2079 (25.8)	597 (20.2)	1482 (29.1)	<0.001	1849 (24.9)	230 (36.9)	<0.001
Abdominal MRI performed for staging, *n* (%)	148 (1.8)	47 (1.6)	101 (2)	0.2	136 (1.8)	12 (1.9)	0.9
PSMA‐PET performed for staging, *n* (%)	400 (5)	89 (3)	311 (6.1)	<0.01	354 (4.8)	46 (7.4)	<0.01

### Use and Diagnostic Performance Characteristics of Staging Procedures for LNI

Overall (Table 1), 25.8% (*n* = 2079), 5% (*n* = 400) and 1.8% (*n* = 148) had an abdominal CT, PSMA‐PET and abdominal MRI for staging, respectively. In patients with NCCN unfavourable IR disease, CT (29.1%) and PSMA‐PET (6.1%) were more frequently performed compared to patients with NCCN favourable risk disease (20.2% CT, 3% PSMA‐PET). Similar rates were recorded for MRI (2% unfavourable vs 1.6% favourable IR, *P* = 0.2). Of the performed stagings only 0.4% (nine) of CTs, 5% (20) of PSMA‐PETs and 0.7% (one) of MRIs, indicated cN1 disease (Table 2).

**Table 2:** Diagnostic accuracy of various imaging modalities for pelvic lymph node staging in patients with IR PCa.

CT			
Overall population, *N* = 2079	pN1, *n* = 230	pN0, *n* = 1849	Prevalence pN1: 11.1%
cN1 *n* = 9	TP *n* = 2	FP *n* = 7	PPV = TP/(TP + FP) 22.2%
cN0 *n* = 2070	FN *n* = 228	TN *n* = 1842	NPV = TN/(FN + TN) 89%
	Sensitivity = TP/(TP + FN) 0.9%	Specificity = TN/(FP + TN) 99.6%	Accuracy = TP + TN/All 88.7%

PSMA‐PET			
Overall population, *N* = 400	pN1, *n* = 46	pN0, *n* = 354	Prevalence pN1: 11.5%
cN1 *n* = 20	TP *n* = 6	FP *n* = 14	PPV = TP/(TP + FP) 30%
cN0 *n* = 380	FN *n* = 40	TN *n* = 340	NPV = TN/(FN + TN) 89.5%
	Sensitivity = TP/(TP + FN) 13.0%	Specificity = TN/(FP + TN) 96.0%	Accuracy = TP + TN/All 86.5%

MRI			
Overall population, *N* = 148	pN1, *n* = 12	pN0, *n* = 136	Prevalence pN1: 8.1%
cN1 *n* = 1	TP *n* = 1	FP *n* = 0	PPV = TP/(TP + FP) 100%
cN0 *n* = 147	FN *n* = 11	TN *n* = 136	NPV = TN/(FN + TN) 92.5%
	Sensitivity = TP/(TP + FN) 8.3%	Specificity = TN/(FP + TN) 100%	Accuracy = TP + TN/All 92.6%

FN, false negative; FP, false positive; TN, true negative; TP, true positive.

Sensitivity (Table 2) in the overall cohort was highest for PSMA‐PET (13%), followed by MRI (8.3%) and lowest for CT (0.9%). The NPV was highest for MRI (92.5%) and similar for PSMA‐PET (89.5%) and CT (89%). Overall accuracy was 92.6%, 88.7% and 86.5% for MRI, CT and PSMA‐PET, respectively. When only patients with NCCN unfavourable IR PCa were observed similar findings were obtained (Table S1). For patients with NCCN favourable IR PCa (Table S2) a lower sensitivity and PPVs throughout the different imaging methods was recorded.

### Logistic Regression Models Predicting LNI

In multivariable logistic regression models (Table 3), a higher ISUP Grade Group, higher clinical stage, higher preoperative PSA, older age at RP, and a higher number of positive cores at biopsy were all independent predictors for LNI.

**Table 3:** Multivariable logistic regression models predicting LNI in patients with IR PCa undergoing RP with PLND.

	Odds ratio (95% CI)	*P*
ISUP Grade Group 1 (reference)	1.00	–
ISUP Grade Group 2	1.95 (1.30–3.03)	<0.01
ISUP Grade Group 3	4.65 (3.10–7.24)	<0.001
cT1 (reference)	1.00	–
cT2a	1.55 (1.26–1.90)	<0.001
cT2b	2.73 (2.10–3.51)	<0.001
PSA (ng/mL)	1.11 (1.09–1.13)	<0.001
Age (years)	1.02 (1.01–1.03)	<0.01
Number of positive cores	1.10 (1.07–1.13)	<0.001

### Oncological Outcomes after RP

**Fig. 2:**
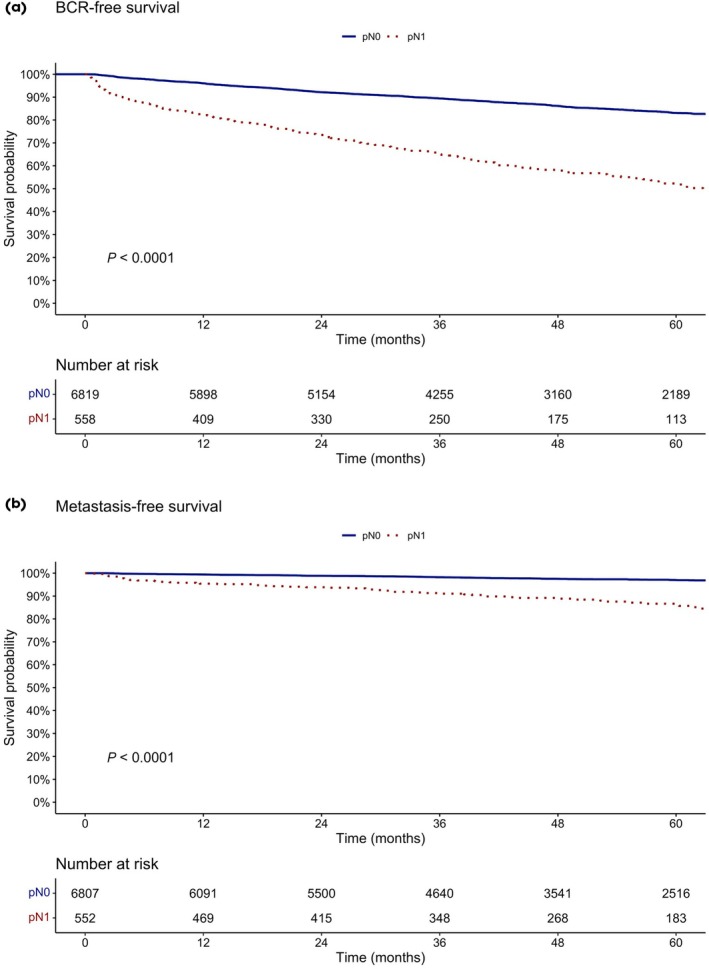
The BCR‐free (**a**) and metastasis‐free (**b**) survival after RP for intermediate‐risk PCa patients stratified according to lymph node negative (pN0) and positive (pN1) disease.

At 60 months after RP, BCR‐free survival was 83.1% vs 52.3% (*P* < 0.001) for pathological N stage (pN)0 vs pN1 (Fig. 2a), respectively. Metastasis‐free survival at 60 months was 97% vs 86.2% (*P* < 0.001) for pN0 vs pN1 patients (Fig. 2b).

**Fig. 3:**
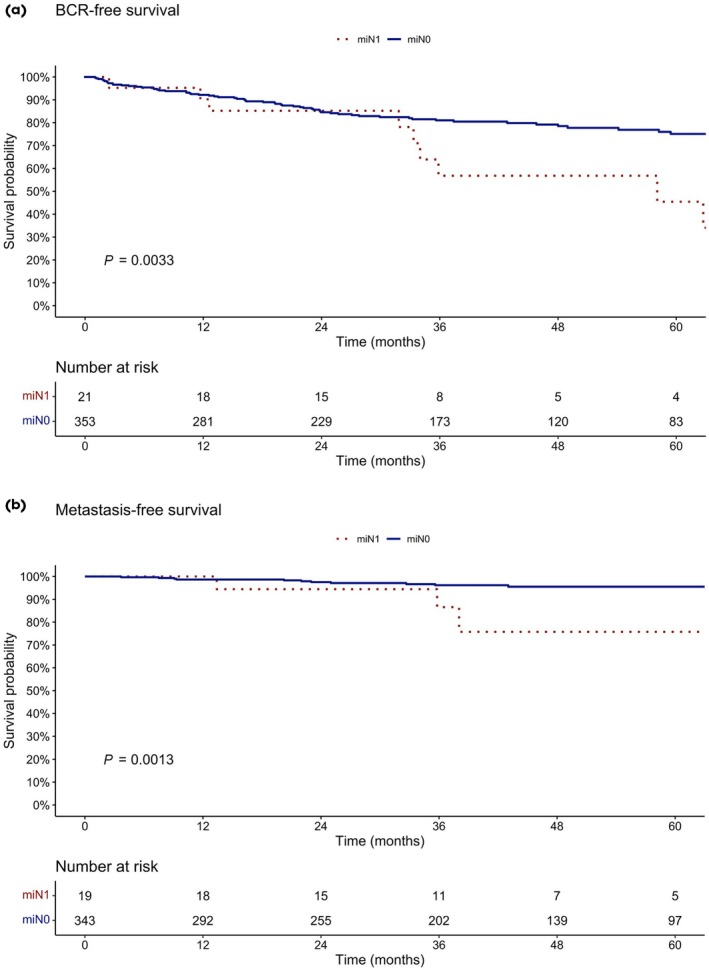
The BCR‐free (**a**) and metastasis‐free (**b**) survival after RP for intermediate‐risk PCa patients stratified according to negative (miN0) and positive (miN1) PSMA‐PET prior to RP.

Patients with suspicious pelvic lymph nodes in PSMA‐PET prior to RP (molecular imaging [mi]N1) had worse BCR‐free survival compared to patients with unsuspicious PSMA‐PET prior to RP (miN0). BCR‐free survival at 60 months (Fig. 3a) was 75.1% vs 45.4% (*P* < 0.01) for miN0 vs miN1 patients, respectively. Similarly, patients with miN1 at PSMA‐PET prior to RP had worse metastasis‐free survival compared to patients with miN0 at PSMA‐PET. Here, metastasis‐free survival at 60 months (Fig. 3b) was 95.5% vs 75.8% (*P* < 0.01) for miN0 vs miN1 patients, respectively.

Similar trends were recorded when patients were stratified into NCCN favourable vs unfavourable IR disease (Figures S1 and S2).

## Discussion

The findings from this large cohort of patients with IR PCa undergoing RP with PLND highlight significant limitations in current preoperative imaging modalities for pelvic lymph node staging. Among >8000 patients, histologically confirmed LNI was present in only 3.2% of favourable and 10.4% of unfavourable NCCN IR disease. However, the performance of commonly used imaging methods—CT, abdominal MRI, and even PSMA‐PET—was suboptimal in this setting, with particularly low sensitivity across all modalities.

Of the three imaging techniques evaluated, PSMA‐PET demonstrated the highest sensitivity (13%) and a relatively favourable NPV of 89.5%. While still limited, these results suggest that PSMA‐PET may identify a subset of patients with nodal metastases that are otherwise occult on conventional imaging. In contrast, CT and MRI performed considerably worse, with sensitivities of 0.9% and 8.3%, respectively. Despite the high NPV for MRI (92.5%) and CT (89%), these values are insufficient to reliably exclude nodal disease, particularly when treatment intensification decisions may hinge on such findings. It is important to note that the NPV is highly dependent on the underlying prevalence of positive lymph nodes. In populations with low event rates, the NPV will naturally be higher, independent of the intrinsic performance of the test. In our study, the distribution of imaging modalities across risk groups was not uniform: PSMA‐PET was more frequently performed in patients with unfavourable IR features, whereas CT and MRI were more evenly distributed across risk groups. Therefore, differences in the NPV between modalities largely reflect the differing prevalence of nodal metastases in the subgroups rather than a true difference in diagnostic accuracy. Prevalence of LNI was 11.1% vs 11.5% vs 8.1% for patients with CT vs PSMA‐PET vs MRI. For this reason, direct comparisons of the NPV between modalities should be interpreted cautiously.

The extremely low true positive rates and high false negative rates across all modalities raise important questions about the current role of imaging in staging IR PCa. CT, historically the most commonly used imaging tool, appears to offer negligible value in this context and may no longer be justified for routine use given its poor sensitivity and associated radiation exposure. Similarly, the limited sensitivity of MRI also calls into question its utility in nodal staging, despite its high NPV.

The PSMA‐PET, though not without limitations, showed relatively better performance, with a small proportion of true positives and fewer false positives compared to CT and MRI similar to a previously published article within a cohort of patients with IR and high‐risk PCa [13]. However, the modest sensitivity still indicates that a significant number of patients with histologically confirmed LNI are not identified preoperatively. This questions the general role of distant staging in patients with IR PCa. Avoiding distant staging in this large patient cohort would also result in a potential large cost reduction for our healthcare systems.

On the other hand, PSMA‐PET was useful in the prediction of oncological outcomes. Patients with negative PSMA‐PET (miN0) had not only superior BCR‐free but also superior metastasis‐free survival compared to patients with suspicious PSMA‐PET (miN1). This result is also in line with previously published findings, where patients with negative PSMA‐PET had better oncological outcomes after RP [6, 14]. Still, the low sensitivity and prevalence of positive PSMA‐PET scans question its routine use in patients with IR features.

Last but not least, in line with several previous published studies, patients with LNI at RP had worse oncological outcomes compared to patients without LNI [14, 15, 16]. However, it is unclear if missing positive lymph nodes, due to sparing patients from PLND, which are then identified due to PSA persistence or BCR, have worse oncological outcomes compared to their counterparts who did undergo PLND upfront. One previous study from our group demonstrated that patients with IR PCa without a PLND, in case of a negative PSMA‐PET, had similar BCR‐free survival compared to those with a PLND and negative PSMA‐PET [17].

Given these findings, a change in clinical practice may be warranted. Conventional imaging modalities—particularly CT—offer little diagnostic value and may be safely abandoned for nodal staging in IR PCa. If imaging is to be pursued (and evidence of absence of nodal involvement is deemed crucial for further treatment), PSMA‐PET may be the preferred modality, especially in centres where it is available and cost‐effective.

This study has several limitations. First, it is retrospective in nature, which may introduce selection bias and limit causal inference. Second, imaging modality use was not standardised and likely varied by patient's and physician's preference, potentially affecting performance comparisons. Third, PSMA‐PET was available only for a subset of patients, potentially reflecting higher‐risk selection within the IR cohort. Information regarding the specific PSMA tracer used was unavailable, which may influence detection rates. Fourth, only patients who underwent RP were included and patients with neoadjuvant androgen deprivation therapy (ADT) were excluded, which may result in a potential selection bias. However, only approximately one out of 20 patients had any form of neoadjuvant ADT (Fig. 1), and excluding these patients probably had no impact on our findings. Typically, patients with IR PCa do not require any neoadjuvant therapy. Unfortunately, detailed information on the size of lymph‐node metastases—either on imaging or on final pathology—was not available in our dataset. As a result, we are unable to determine the distribution of metastasis size, quantify the size differences between true positives and false negatives, or evaluate whether these characteristics varied between CT, MRI, and PSMA‐PET. Finally, variability in RP technique, imaging interpretation, and pathological assessment could influence lymph‐node yield and detection rates, and limited follow‐up restricts evaluation of long‐term oncological outcomes.

In conclusion, while PSMA‐PET shows promise, the overall performance of all evaluated imaging modalities remains poor in detecting pelvic LNI in patients with IR PCa. These results support a more selective and evidence‐based approach to imaging, reserving PSMA‐PET for specific clinical scenarios, while allowing for skipping staging for most patients with IR PCa.

## Funding Information

This research did not receive any specific grant from funding agencies in the public, commercial, or not‐for‐profit sectors.

## Disclosure of Interests

None to declare.

## Financial Disclosures

Felix Preisser certifies that all conflicts of interest, including specific financial interests and relationships and affiliations relevant to the subject matter or materials discussed in the manuscript (e.g., employment/affiliation, grants or funding, consultancies, honoraria, stock ownership or options, expert testimony, royalties, or patents filed, received, or pending), are the following: None.

## Author Contributions

The authors listed below have made substantial contributions to the intellectual content of the paper in the various sections described below. Conceptualisation: Tobias Maurer, Ben Frederik Hartwieg, Christopher Kniep; Methodology: Derya Tilki, Felix Preisser; Data Curation: Ben Frederik Hartwieg, Christopher Kniep; Formal analysis: Felix Preisser, Tobias Maurer, Markus Graefen. Writing ‐ Original Draft: Felix Preisser, Tobias Maurer, Derya Tilki, Markus Graefen, Mike Wenzel, Fabian Falkenbach; Writing – Review and Editing: Philipp Mandel, Tobias Maurer, Alexander Haese, Georg Salomon, Lars Budäus, Thomas Steuber. Visualisation: Felix Preisser, Kristian Krpina; Supervision: Felix Preisser, Tobias Maurer; Funding acquisition: None.

## Supporting information


**Fig. S1.** The BCR‐free (a) and metastasis‐free (b) survival after RP stratified according to pN0 and pN1 disease and favourable vs unfavourable IR disease.

**Fig. S2.** The BCR‐free (a) and metastasis‐free (b) survival after RP stratified according to miN0 and miN1 disease at PSMA‐PET prior to RP and favourable vs unfavourable IR disease.

**Table S1.** Diagnostic accuracy of various imaging modalities for pelvic lymph node staging in patients with unfavourable IR PCa only.

**Table S2.** Diagnostic accuracy of various imaging modalities for pelvic lymph node staging in patients with favourable IR PCa only.
