# Supplementary material for: Diagnostic accuracy and outcomes of lymph node staging in intermediate‐risk prostate cancer
Source: BJU Int. 2026 Jan 23;137(4):667–76. doi: 10.1111/bju.70155 (PMC12962846; doi:10.1111/bju.70155)
Supplement: Supplementary file 1 — Fig. S1. The BCR‐free (a) and metastasis‐free (b) survival after RP stratified according to pN0 and pN1 disease and favourable vs unfavourable IR disease. [file BJU-137-667-s003.docx]

**Supplementary Figure 1**

**a)**

**b)**
