# Supplementary material for: Diagnostic accuracy and outcomes of lymph node staging in intermediate‐risk prostate cancer
Source: BJU Int. 2026 Jan 23;137(4):667–76. doi: 10.1111/bju.70155 (PMC12962846; doi:10.1111/bju.70155)
Supplement: Supplementary file 3 — Table S1. Diagnostic accuracy of various imaging modalities for pelvic lymph node staging in patients with unfavourable IR PCa only. [file BJU-137-667-s002.docx]

**Supplementary Table 1: Diagnostic accuracy of various imaging modalities for pelvic lymph node staging in patients with unfavourable intermediate risk prostate cancer only.**

| CT | | | |
| --- | --- | --- | --- |
| Overall population  n=1482 |  | | Prevalence pN1: 14.0% |
|  | **pN1**  n= 208 | **pN0**  n= 1274 |  |
| **cN1**  n= 9 | TP  n= 2 | FP  n= 7 | PPV  =TP/(TP+FP)  22.2% |
| **cN0**  n= 1473 | FN  n= 206 | TN  n= 1267 | NPV  =TN/(FN+TN)  86% |
|  | Sensitivity  =TP/(TP+FN) 1.0% | Specificity  =TN/(FP+TN) 99.5% | Accuracy  =TP+TN/All 85.6% |

| PSMA | | | |
| --- | --- | --- | --- |
| Overall population  n=311 |  | | Prevalence pN1: 13.5% |
|  | **pN1**  n= 42 | **pN0**  n= 269 |  |
| **cN1**  n= 15 | TP  n= 5 | FP  n= 10 | PPV  =TP/(TP+FP)  33.3% |
| **cN0**  n= 296 | FN  n= 37 | TN  n= 259 | NPV  =TN/(FN+TN)  87.5% |
|  | Sensitivity  =TP/(TP+FN) 11.9% | Specificity  =TN/(FP+TN) 96.3% | Accuracy  =TP+TN/All  84.9% |

| MRI | | | |
| --- | --- | --- | --- |
| Overall population  n=101 |  | | Prevalence pN1: 9.9% |
|  | **pN1**  n= 10 | **pN0**  n= 91 |  |
| **cN1**  n= 1 | TP  n= 1 | FP  n= 0 | PPV  =TP/(TP+FP)  100% |
| **cN0**  n= 100 | FN  n= 9 | TN  n= 91 | NPV  =TN/(FN+TN)  91% |
|  | Sensitivity  =TP/(TP+FN) 10.0% | Specificity  =TN/(FP+TN) 100% | Accuracy  =TP+TN/All 91.1% |

Abbreviations: TP – true positive, FP – false positive, FN –false negative, TN – true negative, PPV –positive predictive value, NPV – negative predictive value.
