# Supplementary material for: Diagnostic accuracy and outcomes of lymph node staging in intermediate‐risk prostate cancer
Source: BJU Int. 2026 Jan 23;137(4):667–76. doi: 10.1111/bju.70155 (PMC12962846; doi:10.1111/bju.70155)
Supplement: Supplementary file 4 — Table S2. Diagnostic accuracy of various imaging modalities for pelvic lymph node staging in patients with favourable IR PCa only. [file BJU-137-667-s004.docx]

**Supplementary Table 2: Diagnostic accuracy of various imaging modalities for pelvic lymph node staging in patients with favourable intermediate risk prostate cancer only.**

| CT | | | |
| --- | --- | --- | --- |
| Overall population  n=597 |  | | Prevalence pN1: 3.7% |
|  | **pN1**  n= 22 | **pN0**  n= 575 |  |
| **cN1**  n= 1 | TP  n= 0 | FP  n= 1 | PPV  =TP/(TP+FP)  0% |
| **cN0**  n= 596 | FN  n= 22 | TN  n= 574 | NPV  =TN/(FN+TN)  96.3% |
|  | Sensitivity  =TP/(TP+FN) 0% | Specificity  =TN/(FP+TN) 99.8% | Accuracy  =TP+TN/All 96.1% |

| PSMA | | | |
| --- | --- | --- | --- |
| Overall population  n=89 |  | | Prevalence pN1: 4.5% |
|  | **pN1**  n= 4 | **pN0**  n= 85 |  |
| **cN1**  n= 5 | TP  n= 1 | FP  n= 4 | PPV  =TP/(TP+FP)  20% |
| **cN0**  n= 84 | FN  n= 3 | TN  n= 81 | NPV  =TN/(FN+TN)  96.4% |
|  | Sensitivity  =TP/(TP+FN) 25% | Specificity  =TN/(FP+TN) 95.3% | Accuracy  =TP+TN/All  92.1% |

| MRI | | | |
| --- | --- | --- | --- |
| Overall population  n=47 |  | | Prevalence pN1: 4.3% |
|  | **pN1**  n= 2 | **pN0**  n= 45 |  |
| **cN1**  n= 0 | TP  n= 0 | FP  n= 0 | PPV  =TP/(TP+FP)  0% |
| **cN0**  n= 47 | FN  n= 2 | TN  n= 45 | NPV  =TN/(FN+TN)  95.7% |
|  | Sensitivity  =TP/(TP+FN) 0% | Specificity  =TN/(FP+TN) 100% | Accuracy  =TP+TN/All 95.7% |

Abbreviations: TP – true positive, FP – false positive, FN –false negative, TN – true negative, PPV –positive predictive value, NPV – negative predictive value.
